# Emergency obstetric and newborn care in conflict-affected Somalia: rural access inequities, referral barriers, and health-system priorities — a policy and practice review

**DOI:** 10.3389/fgwh.2026.1914864

**Published:** 2026-08-13

**Authors:** Abdukadir Mohamed Hassan, Abdiwali Mohamed Hussein, Ahmed Omar Siyad

**Affiliations:** 1 Department of Critical Care Medicine, Dr. Sumait Hospital, SIMAD University, Mogadishu, Somalia; 2 Department of Obstetrics and Gynecology, Dr. Sumait Hospital, SIMAD University, Mogadishu, Somalia

**Keywords:** armed conflict, emergency obstetric and newborn care, health systems, health-system resilience, humanitarian health, maternal health, maternal mortality, neonatal mortality

## Abstract

Emergency obstetric and newborn care remains a critical maternal-health and health-system priority for reducing preventable maternal and neonatal deaths in Somalia. Prolonged conflict, displacement, recurrent climate shocks, poverty, and rural access inequities continue to expose pregnant women and newborns to delays in reaching timely emergency care. Although previous reviews have described broad maternal and newborn health challenges in Somalia, less attention has been given to how emergency obstetric and newborn care gaps interact with rural geography, insecurity, referral barriers, workforce shortages, weak facility readiness, and humanitarian service-delivery constraints. This policy and practice review synthesizes peer-reviewed literature, national survey evidence, policy documents, technical guidance, and authoritative reports to examine emergency obstetric and newborn care in conflict-affected Somalia. Sources published or made available between 2010 and 2026 were prioritized, while older seminal sources were retained only when they provided foundational frameworks or technical definitions. The review focuses on rural access inequities, referral barriers, facility readiness, workforce capacity, sociocultural determinants of care-seeking, and policy-practice priorities. The evidence indicates that low rates of skilled birth attendance, limited institutional delivery, inadequate basic and comprehensive emergency obstetric and newborn care coverage, weak referral systems, financial barriers, and shortages of skilled personnel continue to undermine timely care for obstetric and newborn emergencies. Strengthening emergency obstetric and newborn care in Somalia requires an integrated service-delivery and policy approach that expands rural coverage, improves referral coordination, invests in midwifery and emergency obstetric skills, stabilizes essential supplies and blood transfusion capacity, strengthens maternal and perinatal death surveillance, and aligns humanitarian and development programming. Addressing these priorities is essential for improving equitable maternal and newborn survival in conflict-affected and resource-limited settings.

## Background

Maternal and newborn survival is an urgent maternal-health, health-equity, and health-system priority in Somalia. Globally, an estimated 287,000 women died from maternal causes in 2020, and sub-Saharan Africa accounted for approximately 70% of these deaths, reflecting the persistent concentration of preventable maternal mortality in low-resource and fragile settings (1). Somalia continues to be among the countries with the highest maternal and neonatal mortality burdens. The 2023 United Nations maternal mortality estimates reported a maternal mortality ratio of 621 deaths per 100,000 live births for Somalia in 2020, while Somalia's maternal and newborn health country profile reported neonatal mortality of 36 deaths per 1000 live births and stillbirths of 28 per 1000 births (1, 2). These mortality patterns are particularly concerning because many deaths around childbirth are preventable when skilled birth attendance, timely emergency obstetric and newborn care, referral systems, essential medicines, blood transfusion services, and quality intrapartum care are available (3–5).

Somalia's maternal and newborn health indicators reflect the combined effects of health-system fragility, conflict, rural inaccessibility, socioeconomic deprivation, and uneven service coverage. The Somalia Health and Demographic Survey reported that only 32% of births were attended by skilled health personnel and that 31% of women received antenatal care from a skilled provider during their most recent pregnancy. It also found that 89% of mothers did not receive a postnatal check within the first two days after childbirth (6).

These gaps occur when women and newborns are highly vulnerable to postpartum hemorrhage, hypertensive emergencies, obstructed labor, sepsis, birth asphyxia, complications of prematurity, and severe neonatal infection (3, 7). Low coverage of skilled attendance and institutional delivery is therefore not only a service-utilization problem but also an emergency-care and patient-safety concern.

Emergency obstetric and newborn care is especially important in Somalia because many life-threatening complications are unpredictable and require timely intervention. The World Health Organization (WHO), United Nations Population Fund, UNICEF, and partners define emergency obstetric care through signal functions that indicate whether a facility can provide life-saving interventions for obstetric complications. Basic emergency obstetric and newborn care (BEmONC) includes core functions such as parenteral antibiotics, uterotonics, anticonvulsants, manual removal of the placenta, removal of retained products, assisted vaginal delivery, and basic newborn resuscitation, while comprehensive emergency obstetric and newborn care (CEmONC) adds surgical capacity and blood transfusion (3). In conflict-affected and rural settings, the absence of these functions can turn otherwise treatable complications into fatal events. This makes emergency obstetric and newborn care (EmONC) a central measure of both women's health equity and health-system resilience.

Somalia's EmONC gaps are strongly shaped by geography and insecurity. National readiness evidence documents a pronounced urban–rural disparity in emergency maternal and newborn services (8). This disparity helps explain why rural women, nomadic populations, internally displaced persons, and communities in insecure districts may experience dangerous delays in reaching functional care. Somalia's national Reproductive, Maternal, Newborn, Child and Adolescent Health (RMNCAH) policy brief also identifies governance and implementation constraints that compound geographic inequities (7).

The referral system is another critical weakness. Somalia's Essential Package of Health Services (EPHS) implementation strategy recognizes that effective referral is essential for access to BEmONC and CEmONC and calls for locally feasible transport and financing arrangements (9). This is highly relevant where emergency transport is limited, routes may be unsafe, and households face out-of-pocket costs. The three-delays framework remains useful because deaths may occur when families delay seeking care, transport or insecurity delays arrival, or facilities delay recognition and treatment (10).

Recent Somalia-specific studies reinforce the importance of linking service coverage with access, knowledge, autonomy, and facility readiness. A cross-sectional study on antenatal care utilization found that family influence, women's autonomy, and knowledge of midwife availability were associated with antenatal care attendance and subsequent institutional delivery (11). An analysis of Somalia's 2020 Health and Demographic Survey reported very low completion of the maternity continuum of care, showing that women often fail to receive a complete sequence of antenatal care, skilled birth attendance, and postnatal care (12). Another national analysis reported that only 7% of pregnancies received the minimum of four antenatal care visits, with rural and nomadic women facing substantial disadvantage (13). Studies of institutional and facility delivery also show that financial constraints, distance, low awareness, poor antenatal care uptake, nomadic residence, high parity, early marriage, and perceived distance to health facilities are associated with home delivery or lower facility delivery utilization (14, 15).

A recent broad narrative review synthesized maternal and newborn health in Somalia across mortality, service coverage, sociocultural determinants, and general public-health priorities (16). A single Somalia case report also described one maternal death following conflict-related facility closure and delayed referral; this case is illustrative and cannot establish the frequency or national pattern of referral failure (17). A more focused synthesis is therefore needed to examine EmONC as an emergency-care pathway linking community recognition, referral, stabilization, definitive treatment, and preventable mortality in rural and conflict-affected settings.

The present review differs from Mohamoud et al. (16) in four specific respects. First, EmONC functionality—not maternal and newborn health generally—is the organizing unit of analysis. Second, the review applies the three-delays framework to connect household decision-making, transport, and facility response. Third, it distinguishes nominal facility availability from performance of BEmONC and CEmONC signal functions. Fourth, it translates the evidence into operational priorities for geographic coverage, referral coordination, workforce readiness, essential inputs, community linkage, digital support, and accountability. This narrower pathway-based analysis is the review's principal contribution.

The aim of this policy and practice review is to synthesize available evidence on emergency obstetric and newborn care in conflict-affected Somalia, with emphasis on rural access inequities, referral barriers, facility readiness, workforce constraints, and health system priorities. The review seeks to support policy makers, health leaders, clinicians, midwives, public health professionals, and humanitarian partners in designing context-specific strategies to improve timely, safe, and equitable care for women and newborns.

## Methods: narrative review approach

This study used a narrative review design to synthesize evidence on emergency obstetric and newborn care in Somalia. A narrative approach was selected because the available evidence is heterogeneous and includes national surveys, observational studies, policy briefs, humanitarian reports, health system strategy documents, and global guidance rather than a single body of comparable clinical trials or quantitative studies. The review was designed to provide an integrated, policy-relevant synthesis rather than a meta-analysis.

A structured literature search was conducted from May 1 to May 11, 2026, for evidence available up to May 2026. The 11-day period represents the active retrieval and source-checking period, not the publication window. Searches were conducted sequentially across PubMed, Google Scholar, Dove Medical Press, Frontiers, PLOS Global Public Health, WHO, UNICEF, UNFPA, Healthy Newborn Network, and Somalia Ministry of Health sources. Peer-reviewed articles, national reports, policy documents, and technical guidance published or made available between 2010 and 2026 were prioritized. Older seminal sources were retained only for established frameworks or technical definitions, including the Three Delays Model and EmONC signal-function monitoring. Search terms combined ’Somalia' with ‘emergency obstetric care,’ ‘emergency obstetric and newborn care,’ ‘BEmONC,’ ‘CEmONC,’ ‘maternal mortality,’ ‘neonatal mortality,’ ’skilled birth attendance,’ ‘institutional delivery,’ ‘facility delivery,’ ‘rural access,’ ‘nomadic populations,’ ‘referral system,’ ‘conflict,’ ‘displacement,’ ‘RMNCAH,’ ‘risk management,’ ‘healthcare policy,’ and ‘health system strengthening.’ Grey literature was included because much Somalia-specific EmONC evidence is reported through national surveys, strategies, country-office materials, and policy briefs. Only English-language sources were reviewed.

Documents were considered for inclusion when they had direct relevance to Somalia or provided authoritative global guidance applicable to emergency obstetric and newborn care in low-resource, rural, fragile, or conflict-affected settings. Somalia-specific sources were prioritized when they reported maternal or neonatal mortality, antenatal care, skilled birth attendance, institutional or facility delivery, rural or nomadic access, referral systems, EmONC availability, workforce capacity, facility readiness, or RMNCAH policy priorities. Global sources were included when they defined EmONC, described effective interventions, or provided relevant frameworks for maternal mortality reduction, intrapartum care, antenatal care, quality health systems, community-based interventions, protection of health services in conflict, or maternal and perinatal death surveillance.

One author led the searches, assessed source relevance, and conducted the initial evidence extraction. The other two authors critically reviewed the synthesis and use of sources before submission. Because this was planned as a narrative rather than systematic review, total initial search hits and deduplicated records were not prospectively logged; a reliable approximate initial-record count cannot therefore be reconstructed. The initial submission included 24 sources. Three sources were added in response to peer review, giving 27 sources in the revised synthesis. Evidence was organized into five analytic domains: mortality burden and preventability, rural access inequities, referral barriers, facility readiness and workforce capacity, and risk-management and health-policy priorities. These evidence domains informed seven action-oriented policy priorities; mobile health/telehealth and data/accountability were treated as cross-cutting implementation priorities rather than additional evidence domains. No formal meta-analysis or risk-of-bias assessment was performed. Triangulation across peer-reviewed evidence, national surveys, WHO and Ministry of Health documents, and Somalia-specific policy materials was used to improve contextual relevance.

The included peer-reviewed articles, national reports, policy documents, and technical guidance materials are summarized in Supplementary Table S1.

## Emergency obstetric and newborn care in Somalia's fragile health system

Emergency obstetric and newborn care is a foundational intervention for reducing maternal and neonatal mortality because complications during pregnancy, childbirth, and the immediate postnatal period can progress rapidly and require timely clinical response (3–5). The classic evidence base on maternal mortality reduction emphasizes that skilled attendance at birth must be linked to functioning referral and emergency obstetric care, because the survival benefit depends not only on the presence of a birth attendant but also on the ability to manage hemorrhage, eclampsia, infection, obstructed labor, fetal distress, and neonatal compromise (4). The Lancet series on maternal and newborn survival further showed that proven interventions can prevent large numbers of maternal deaths, newborn deaths, and stillbirths when delivered with sufficient coverage and quality (5).

In Somalia, the relevance of EmONC is heightened by persistent gaps in facility delivery and skilled attendance documented in national and WHO evidence (6, 8). Many women therefore give birth outside settings capable of providing timely emergency care. Because obstetric complications cannot be reliably predicted before labor, risk screening alone cannot substitute for access to functioning emergency services (3, 4).

Somalia's fragile health-system context further compounds EmONC gaps. National policy evidence indicates that rural and hard-to-reach areas face particularly limited workforce coverage and implementation capacity (7). The EPHS implementation strategy also notes that it has not always been possible to guarantee the 24-hour staffing needed for sufficient BEmONC and CEmONC provision (9). Consequently, reaching a facility does not ensure that definitive care will be available at all hours.

The distinction between nominal service availability and actual emergency functionality is important. EmONC is not defined merely by the existence of a health facility, a maternity ward, or a trained provider. It depends on whether signal functions have actually been performed within a defined period and whether facilities have the drugs, equipment, blood transfusion capacity, surgical capacity, infection prevention systems, newborn resuscitation capacity, and referral linkages needed to respond to emergencies (3). In Somalia, where facilities may face insecurity, workforce shortages, supply interruptions, and dependence on humanitarian support, measuring actual EmONC functionality is essential for identifying where women and newborns remain at highest risk.

## Rural access inequities and conflict-related barriers

Rural access inequity is one of the most important dimensions of emergency maternal and newborn care in Somalia. WHO Somalia reported that BEmONC services were available in 45% of urban health facilities compared with only 20% of rural health facilities in the 2016 SARA assessment (8). This urban-rural gap is clinically significant because emergency obstetric complications require timely intervention, and rural women may face long distances, poor roads, seasonal barriers, limited transport, insecurity, and cost-related delays before reaching care. The same WHO report indicated low facility delivery and skilled provider support nationally, reinforcing the concern that emergency care access remains structurally limited (8).

Conflict and insecurity intensify rural access barriers by disrupting mobility, damaging infrastructure, constraining health worker deployment, weakening supply chains, and reducing household confidence in traveling to facilities during labor or emergencies (7, 18). Insecure roads, checkpoints, and unpredictable violence can turn geographic distance into a life-threatening barrier. These conditions are particularly harmful for women in labor because the window for managing hemorrhage, eclampsia, obstructed labor, sepsis, or fetal compromise may be short. Conflict also contributes to displacement, and displaced women may live in settlements where health services depend heavily on humanitarian agencies, mobile clinics, or temporary delivery points that may not have full EmONC capacity (7, 9).

Nomadic and rural populations face additional structural disadvantages. A national analysis of antenatal care utilization found that only 7% of pregnancies received the minimum of four antenatal care visits, with rural and nomadic women having markedly lower odds of adequate antenatal care than urban women (13). Facility delivery research using Somalia's 2020 demographic survey data similarly found that nomadic residence and perceived distance to the nearest facility were associated with lower likelihood of facility-based delivery (15). These findings suggest that the same populations least likely to receive antenatal care and facility delivery are also the populations most likely to face delays when emergency obstetric or newborn complications arise.

Rural inequity is not only geographic but also financial, informational, and sociocultural. Studies from Somalia have shown that family influence, women's autonomy, knowledge of midwife availability, lack of understanding about the importance of facility delivery, financial constraints, and distance influence maternal health service utilization (11, 14, 15). In major Somali towns, where facilities are more concentrated than in rural settings, home delivery remains common and is associated with poor knowledge of facility delivery, non-use of antenatal care, lack of counseling on place of delivery, financial reasons, and distance to facilities (14). If women in urban areas still face these barriers, the burden is likely more severe in rural and conflict-affected settings where facilities and transport are more limited.

National synthesis also risks obscuring regional heterogeneity. Somaliland, Puntland, and South-Central Somalia differ in governance arrangements, security conditions, partner presence, infrastructure, workforce distribution, and the continuity of health services (6, 7, 9). Consequently, national and urban–rural averages should not be assumed to describe every region or district. EmONC mapping and referral design should therefore use subnational data and locally validated service pathways wherever possible.

## Referral barriers and the three delays in emergency care

Referral is the central link between community-level maternal risk and facility-based emergency care. In Somalia, referral weakness is repeatedly identified as a health system barrier. The Essential Package of Health Services implementation strategy explicitly states that an effective referral system is essential to ensure access to basic and comprehensive emergency obstetric and newborn care, and it recognizes the need to analyze current referral models, including ambulances, community cost-sharing, donkey carts, TukTuk ambulances, conditional cash transfers for local transport, and flexible contracting arrangements for service providers (9). This policy recognition is important because Somalia's rural and conflict-affected settings require referral solutions that are both clinically reliable and locally feasible.

The three-delays framework provides a useful structure for interpreting referral barriers in Somalia. The first delay occurs when a woman or family delays the decision to seek care because of limited awareness of danger signs, low perceived risk, sociocultural norms, lack of decision-making autonomy, fear of costs, or preference for home delivery (10, 11, 14). The second delay occurs when the woman decides to seek care but cannot reach a facility quickly because of distance, insecurity, transport scarcity, road conditions, cost, or weak ambulance systems (8, 10, 14, 15). The third delay occurs after arrival when the facility cannot provide prompt, high-quality care because of staff shortages, stock-outs, lack of blood transfusion, limited surgical capacity, delayed triage, or weak newborn resuscitation readiness (7–9).

Referral barriers are especially dangerous for obstetric emergencies because the clinical time available for intervention can be very short. Postpartum hemorrhage, eclampsia, obstructed labor, ruptured uterus, neonatal asphyxia, and severe newborn infection require early recognition, stabilization, and transfer when higher-level care is needed (3, 19). A referral system that depends on informal transport, household payment, or *ad hoc* communication between facilities may fail during precisely the moments when rapid response is most necessary. Therefore, strengthening EmONC in Somalia requires referral pathways that include community recognition of danger signs, pre-arranged transport options, communication between facilities, stabilization before transfer, clear destination mapping, and accountability for referral completion.

Referral must also be integrated with primary health care and community health systems. The EPHS implementation strategy emphasizes strengthening links between community-based health care and other EPHS service delivery levels to ensure continuity of care (9). This linkage is particularly important for rural and nomadic communities, where women may first interact with female health workers, community health workers, traditional birth attendants, local leaders, or family decision-makers rather than facility-based clinicians. In such settings, community-level actors should be trained to recognize danger signs, promote birth preparedness, encourage antenatal care, support timely referral, and avoid delaying transfer during obstetric and newborn emergencies.

## Facility readiness, workforce capacity, and quality of care

The availability of a facility does not guarantee emergency readiness. High-quality EmONC requires skilled providers, functioning equipment, essential medicines, blood transfusion where comprehensive care is provided, newborn resuscitation capacity, infection prevention, surgical capability when indicated, reliable power and water, clinical protocols, triage systems, and referral communication (3, 20). In Somalia, inconsistent 24-hour staffing and gaps in essential inputs and coordination can prevent facilities from delivering these functions reliably (7, 9). The practical consequence is delayed or incomplete treatment of hemorrhage, eclampsia, obstructed labor, and neonatal compromise.

Workforce capacity is especially central. WHO Somalia has supported development of a standardized BEmONC training package for midwives and nursing staff to manage major causes of maternal and newborn mortality, including hemorrhage, infection, pre-eclampsia and eclampsia, obstructed labor, and newborn asphyxia (8). This training focus is appropriate because nurses, midwives, and frontline maternity providers are often the first and sometimes only skilled personnel available in low-resource districts. However, training alone is insufficient if providers lack essential medicines, supervision, functioning equipment, transport support, referral authority, and safe working conditions. In fragile systems, competency-building must be paired with service readiness and leadership accountability.

Quality of care is also a major determinant of survival. The Lancet Global Health Commission on high-quality health systems emphasized that mortality reduction depends not only on access to services but also on competent systems that consistently provide effective, respectful, and timely care (20). This is particularly relevant in Somalia, where increasing facility delivery without improving readiness could shift women into facilities that remain unable to manage complications. A high-quality EmONC agenda should therefore include clinical standards, respectful maternity care, emergency drills, audit and feedback, maternal and perinatal death reviews, supportive supervision, infection prevention, and continuous monitoring of signal function performance.

Maternal and perinatal death surveillance and response (MPDSR) is an important mechanism for improving quality and accountability. Somalia's RMNCAH policy brief identifies the lack of an MPDSR system as a barrier to evidence-based decision-making and strategic planning (7). WHO guidance emphasizes that MPDSR can help identify causes, avoidable factors, and corrective actions to prevent similar deaths (21). In Somalia, MPDSR should be adapted for fragile and insecure settings and linked to district-level quality improvement, referral review, and facility-readiness planning.

## Policy and practice priorities for strengthening EmONC in Somalia

Strengthening EmONC in Somalia requires a health-system, service-delivery, and policy approach rather than isolated clinical training or facility upgrades. The first priority is to expand functional BEmONC and CEmONC coverage in rural, nomadic, and conflict-affected districts. This requires mapping facilities by actual signal-function performance, identifying geographic gaps, and prioritizing underserved regions where travel time to emergency care is longest. The WHO EmONC handbook recommends monitoring availability, geographic distribution, use, met need, cesarean delivery, and quality indicators to assess whether emergency obstetric services are functional and equitably distributed (3). Somalia's EmONC planning should use these indicators to move beyond counting facilities toward measuring life-saving capacity and identifying preventable delay points in the care pathway.

The second priority is to strengthen referral systems for obstetric and newborn emergencies. Building on the context-specific models identified in the EPHS strategy, districts should establish clear communication channels, referral criteria, transport arrangements, stabilization protocols, and feedback loops between lower-level facilities and referral hospitals (9). Plans for rural and insecure areas should account for seasonal road conditions, security risks, community transport assets, and affordability. Monitoring should assess referral initiation, completion, travel time, condition on arrival, and access to definitive care.

The third priority is to invest in the maternal and newborn workforce. Somalia needs sufficient numbers of midwives, nurses, maternity clinicians, anesthetic providers, surgical teams, and newborn care providers who can deliver emergency care safely. Standardized BEmONC training supported by WHO and the Federal Ministry of Health is a positive step, particularly because it targets major causes of maternal and newborn mortality (8). However, workforce policy should also address retention in rural areas, supportive supervision, mentorship, safe working environments, referral decision-making authority, and continuous professional development. Rural EmONC readiness cannot be achieved if trained providers remain concentrated in urban centers.

The fourth priority is to improve facility readiness through reliable supplies, equipment, blood transfusion systems, infection prevention, and emergency protocols. Stock-outs of uterotonics, anticonvulsants, antibiotics, resuscitation equipment, intravenous fluids, and surgical supplies can undermine emergency care even when staff are present (3, 7, 9). CEmONC strengthening must also include safe surgical capacity and blood transfusion, because cesarean delivery and transfusion are defining components of comprehensive emergency care (3). In Somalia, supply chain resilience should be integrated with humanitarian preparedness, because conflict, drought, flooding, and displacement can rapidly increase demand while disrupting logistics.

The fifth priority is to address community-level and sociocultural barriers to timely care. Evidence from Somalia shows that family influence, women's autonomy, knowledge of midwife availability, antenatal care exposure, counseling on delivery location, distance, and financial barriers influence maternal care-seeking (11, 14, 15). Community engagement should therefore focus on birth preparedness, recognition of danger signs, respectful and culturally appropriate counseling, early antenatal contact, and linkage to referral services. Community-based packages can improve maternal and neonatal outcomes when they combine education, mobilization, and connections to appropriate care (22, 23). Male and family engagement may be helpful, but interventions must protect women's autonomy and avoid reinforcing male control over care-seeking (24).

The sixth priority is to use mobile health and telehealth selectively to strengthen—not replace—the EmONC pathway. Somalia's health strategy supports expansion of e-health and telemedicine, while evidence from low- and middle-income countries suggests that mobile-health interventions can improve selected maternal and newborn service-use outcomes, although effects vary by intervention and setting (25, 26). Feasible applications include toll-free danger-sign advice, appointment and birth-preparedness reminders, pre-referral communication, remote consultation between rural teams and referral hospitals, confirmation of bed or blood availability, follow-up after discharge, and distance-based mentoring of frontline staff. Implementation should begin with small, evaluated pilots integrated into district referral protocols and should use low-bandwidth, multilingual platforms with offline contingencies. Data protection, informed consent, equitable access for women without personal phones, network and electricity interruptions, clinical accountability, and the risk of delaying urgent transfer require explicit safeguards. Telehealth must never substitute for emergency transport, physical examination, medicines, blood transfusion, surgery, or newborn resuscitation.

The seventh priority is to strengthen data systems and accountability. Somalia needs more reliable facility reporting, EmONC signal-function monitoring, referral-completion tracking, maternal and perinatal death reviews, and district-level performance dashboards. MPDSR can help identify preventable factors in deaths and convert mortality data into corrective action (21). Data systems should be designed for use by health leaders and facility teams, not only for reporting to donors. In fragile settings, simple indicators that are regularly reviewed and acted upon may be more useful than complex systems that cannot be maintained.

## Discussion

This narrative review highlights that emergency obstetric and newborn care is a critical but underdeveloped pathway for improving maternal and newborn survival and strengthening maternal-health service delivery in Somalia. The evidence suggests that Somalia's high maternal and neonatal mortality burden is not only the result of low service utilization but also reflects deeper emergency care gaps, including limited rural BEmONC availability, weak referral systems, shortages of skilled providers, inadequate facility readiness, and structural barriers related to conflict and poverty (2, 3, 6–9). The problem is therefore clinical, systemic, and directly relevant to maternal health equity, service provision, resource allocation, humanitarian programming, and health policy. Women and newborns need timely access to competent emergency services, but those services must be geographically reachable, financially accessible, culturally acceptable, and functionally ready.

The review also shows that rural inequity is central to the EmONC challenge. Rural and nomadic women are less likely to receive adequate antenatal care, skilled delivery, and facility-based childbirth, and rural facilities are less likely to have BEmONC capacity (8, 13, 15). This creates a compounding risk pathway in which the women most likely to face access barriers are also the least likely to be connected to the health system before labor and the least likely to reach emergency services during complications. For Somalia, equity-focused EmONC planning should therefore prioritize underserved districts rather than only strengthening referral hospitals in urban centers.

Referral barriers deserve special attention because they connect community, transport, primary care, and hospital-level emergency response. The EPHS implementation strategy's recognition of context-specific referral models is important because Somalia's geography and insecurity require flexible solutions (9). Referral strengthening must extend beyond transport availability to include early decision-making, communication and stabilization before transfer, financial protection, and review of failed referrals. Appropriately designed mobile communication and teleconsultation may support these functions, but they cannot compensate for an unavailable vehicle or a receiving facility without definitive-care capacity (25, 26). In parallel, antenatal care contacts should support birth preparedness, early identification of risk, and timely linkage to emergency referral in accordance with WHO recommendations (27).

A key contribution of this review is its distinction from broad maternal and newborn health reviews. A recent Somalia-focused narrative review described Somalia's broader maternal and newborn health challenges, including mortality, service coverage, determinants, and public health priorities (16). The present review narrows the focus to EmONC and examines how emergency care gaps interact with rural access, conflict, referral barriers, and facility readiness. This focused lens is important for policy because many maternal and newborn deaths occur around labor, birth, and the first 24 h, when timely emergency response has the greatest potential to save lives (3, 8).

The findings have important implications for healthcare leadership. Health leaders in Somalia and partner organizations should treat EmONC as a core health system performance indicator and not only as a maternity program component. District and regional managers should know which facilities are performing BEmONC and CEmONC signal functions, which communities face the longest referral times, which facilities lack 24-hour staffing, and which maternal or perinatal deaths were linked to avoidable referral or treatment delays. Leadership should also integrate humanitarian response with longer-term health system strengthening, because Somalia's maternal and newborn survival challenges are both acute and structural (7, 9).

The findings also have implications for clinical practice. Midwives, nurses, obstetric clinicians, anesthetic providers, and newborn care providers require standardized emergency protocols, simulation-based practice, supportive supervision, and reliable supplies. WHO Somalia's national BEmONC training initiative is therefore highly relevant, but its impact will depend on whether trained providers are supported within functional facilities (8). Quality improvement should focus on the complications most likely to cause death, including postpartum hemorrhage, hypertensive emergencies, sepsis, obstructed labor, and neonatal asphyxia (3, 8).

Future research should move beyond descriptive national coverage and examine actual EmONC functionality, referral effectiveness, quality of care, and intervention outcomes at district and regional levels. Somalia-specific evidence remains limited on signal-function performance, travel time, blood transfusion capacity, cesarean readiness, newborn resuscitation quality, referral completion, and death-review implementation. Comparative studies across Somaliland, Puntland, and South-Central Somalia could identify how governance, security, and service-delivery arrangements shape access and outcomes. Implementation research is also needed to test rural referral models, community transport, maternity waiting approaches, mobile-health and telehealth models, workforce-retention strategies, and integrated humanitarian-development approaches. Digital interventions should be evaluated for feasibility, safety, equity, cost, referral timeliness, and clinical outcomes rather than adoption alone.

### Limitations

This review has several limitations. As a narrative review, it did not use a registered systematic protocol, exhaustive database strategy, prospective retrieval log, formal risk-of-bias assessment, or meta-analysis; one author led source screening and selection, and selection bias may therefore be present. Somalia-specific EmONC evidence is sparse, and References 4–7 carry substantial interpretive weight because they provide the principal national policy, readiness, signal-function, and service-delivery evidence. Reliance on this small group of policy and technical sources increases vulnerability to outdated estimates, incomplete geographic coverage, and institutional reporting bias. Several other sources are policy reports or humanitarian documents rather than peer-reviewed studies. Conflict-affected districts may be underrepresented, and national or urban–rural summaries may conceal important differences across Somaliland, Puntland, and South-Central Somalia. Reference 24 is a case report authored by two authors of this review; it is used only as a transparent, illustrative example and not as evidence of prevalence or a national pattern. Direct Somalia-specific evidence on telehealth for obstetric and newborn emergencies is also sparse, so digital-health recommendations draw partly on national strategic direction and broader evidence from low- and middle-income settings and require local evaluation. Despite these limitations, the review triangulates national surveys, Ministry of Health materials, WHO reports, global technical guidance, and recent peer-reviewed studies to provide a focused and policy-relevant synthesis of EmONC access, referral, readiness, and accountability.

## Conclusion

Emergency obstetric and newborn care should be treated as a central maternal-health, equity, and health-system priority in conflict-affected Somalia. The evidence synthesized in this review shows that rural access inequities, conflict-related insecurity, weak referral systems, limited facility readiness, workforce shortages, financial barriers, and sociocultural constraints continue to delay life-saving care for women and newborns. A focused EmONC agenda can move Somalia beyond broad maternal and newborn health planning toward practical action across the care pathway, including community recognition of danger signs, timely referral, functional facility readiness, and accountable quality improvement. Strengthening BEmONC and CEmONC coverage in rural and underserved areas, investing in midwives and frontline providers, improving transport and referral systems, ensuring essential medicines and blood transfusion capacity, and institutionalizing maternal and perinatal death surveillance are essential actions for health-system resilience. Progress will require coordinated leadership from the Federal Ministry of Health, regional authorities, professional associations, communities, and humanitarian and development partners. With sustained investment and context-specific implementation, Somalia can reduce preventable emergency-care delays and improve equitable survival for mothers and newborns.

## Data Availability

No primary data were generated or analyzed for this narrative review. The manuscript is based on published peer-reviewed literature, publicly available national survey findings, policy documents, and reports from authoritative organizations.
