# Supplementary material for: Emergency obstetric and newborn care in conflict-affected Somalia: rural access inequities, referral barriers, and health-system priorities — a policy and practice review
Source: Front Glob Womens Health. 2026 Aug 13;7:1914864. doi: 10.3389/fgwh.2026.1914864 (PMC13518568; doi:10.3389/fgwh.2026.1914864)
Supplement: Supplementary file 1 [file Table1.docx]

**Supplementary Table S1. Summary of Included Evidence, Risks, and Policy Implications for Emergency Obstetric and Newborn Care in Somalia**

| **No.** | **Source (first author/organization, year)** | **Maternal-health, service-delivery, or policy focus** | **Key challenge or system barrier** | **Policy or practice implication for Somalia** |
| --- | --- | --- | --- | --- |
| 1 | World Health Organization, UNICEF, UNFPA, World Bank Group, UNDESA, 2023 | Global maternal mortality surveillance | Preventable maternal mortality remains concentrated in low-resource and fragile settings. | Apply mortality trends and SDG targets to prioritize EmONC coverage, referral strengthening, and preventable-death reduction. |
| 2 | EQUAL Research Consortium / Healthy Newborn Network, 2024 | Somalia maternal and newborn country profiling | High neonatal mortality, stillbirth burden, and gaps in maternal-newborn survival. | Target newborn resuscitation, quality intrapartum care, and facility readiness in high-risk Somali settings. |
| 3 | Directorate of National Statistics, Federal Government of Somalia, 2020 | National survey measurement of service coverage | Low skilled birth attendance, limited ANC coverage, and inadequate postnatal care. | Strengthen continuum-of-care planning and prioritize rural, nomadic, and underserved populations. |
| 4 | WHO Regional Office for the Eastern Mediterranean, 2023 | RMNCAH policy and system strengthening | Workforce shortages, unequal facility distribution, supply gaps, and weak MPDSR. | Expand EPHS coverage, strengthen workforce deployment, and institutionalize maternal and perinatal death review. |
| 5 | WHO, UNFPA, UNICEF, AMDD Program, 2009 | Foundational EmONC signal-function monitoring | Facility availability may not reflect actual emergency obstetric and newborn care functionality. | Use BEmONC and CEmONC signal-function indicators to assess readiness, service functionality, and geographic coverage. |
| 6 | WHO Regional Office for the Eastern Mediterranean, 2023 | BEmONC training and service readiness | Urban-rural BEmONC gaps and inadequate emergency obstetric and newborn capacity. | Scale up competency-based BEmONC training and prioritize rural facility readiness. |
| 7 | Federal Ministry of Health and Human Services, Somalia, 2024 | EPHS implementation and referral planning | Referral weakness, transport constraints, staffing limitations, and fragmented service models. | Formalize referral pathways, transport models, and 24-hour emergency care arrangements. |
| 8 | Thaddeus and Maine, 1994 | Foundational Three Delays framework | Delays may occur in deciding to seek care, reaching care, and receiving adequate facility care. | Design risk-reduction strategies across community decision-making, transport access, referral coordination, and facility response. |
| 9 | Miikkulainen et al, 2023 | Antenatal care utilization and determinants | Family influence, limited autonomy, and incomplete knowledge of midwife availability. | Strengthen community education, women’s decision-making support, and linkage to skilled providers. |
| 10 | Mohamed et al, 2025 | Maternity continuum-of-care analysis | Incomplete progression from ANC to skilled delivery and postnatal care. | Integrate ANC, delivery, postnatal care, and referral systems into a coherent continuum-of-care pathway. |
| 11 | Abdi et al, 2025 | ANC utilization in Somalia | Very low ANC completion and rural or nomadic inequities. | Use outreach, mobile strategies, and equity-focused service planning for rural and nomadic communities. |
| 12 | Sheikh et al, 2024 | Institutional delivery in major towns | Home delivery, limited knowledge, lack of counseling, financial barriers, and distance. | Improve ANC counseling, birth preparedness, transport support, and affordability of facility delivery. |
| 13 | Osman et al, 2026 | Facility delivery determinants | Nomadic residence, perceived distance, parity, wealth, and ANC exposure affect facility delivery. | Target facility delivery interventions to high-risk groups and improve access for nomadic and poor households. |
| 14 | Campbell and Graham, 2006 | Maternal survival strategy | Skilled attendance alone is insufficient without functioning emergency obstetric care. | Link skilled birth attendance to reliable referral pathways and emergency obstetric care capacity. |
| 15 | Bhutta et al, 2014 | Evidence-based maternal and newborn interventions | Low coverage of proven interventions contributes to preventable maternal and newborn deaths. | Scale up high-impact maternal, newborn, and stillbirth prevention interventions with quality and equity. |
| 16 | Mohamoud et al, 2026 | Broad Somalia maternal and newborn health narrative review | Evidence remains fragmented across broad health-system challenges. | Position the present review as a focused synthesis of EmONC risk, access inequity, referral barriers, and policy priorities. |
| 17 | Kruk et al, 2018 | High-quality health-systems framework | Service access without quality may fail to reduce mortality. | Measure quality, readiness, and effective coverage rather than service availability alone. |
| 18 | World Health Organization, 2016 | Antenatal care guideline | Missed opportunities for early risk detection and birth preparedness. | Strengthen ANC contacts, danger-sign counseling, and referral linkage to EmONC services. |
| 19 | World Health Organization, 2018 | Intrapartum care guideline | Weak labor monitoring and poor-quality childbirth care. | Standardize intrapartum care, respectful maternity care, and early complication recognition. |
| 20 | World Health Organization, 2021 | MPDSR implementation guidance | Weak mortality surveillance limits learning from preventable deaths. | Use MPDSR to identify avoidable factors and drive district-level quality improvement. |
| 21 | Lassi and Bhutta, 2015 | Community-based intervention packages | Weak community linkage, delayed care-seeking, and low service uptake. | Strengthen community mobilization, health education, and referral linkage to facility-based care. |
| 22 | Yargawa and Leonardi-Bee, 2015 | Male involvement and maternal health outcomes | Household decision-making barriers and limited support for care-seeking. | Use culturally appropriate family engagement while protecting women’s autonomy. |
| 23 | Rubenstein and Bittle, 2010 | Protection of health care in armed conflict | Insecurity disrupts health workers, facilities, and service continuity. | Protect health workers and facilities and maintain emergency maternal and newborn services during insecurity. |
| 24 | Hassan and Hussein, 2026 | Somalia case report of maternal death in transit | A single reported maternal death illustrates how conflict-related facility closure and delayed transfer can contribute to death in transit; it does not establish prevalence. | Use this case only as contextual illustration when designing referral-continuity and service-protection strategies; population-level inference requires broader evidence. |
| 25 | Foster-Pagaebi et al., 2025 | Community-based healthcare systems and health equity | Weak community engagement and poor linkage between households and appropriate services can sustain inequity. | Combine health education and community mobilization with reliable referral and linkage to facility-based care. |
| 26 | World Health Organization, 2022 | WHO–Somalia cooperation strategy and digital transformation | Geographic isolation, insecurity, and weak information systems constrain access and coordination. | Pilot low-bandwidth e-health and telemedicine within referral pathways, with governance, equity, and data-protection safeguards. |
| 27 | Lee et al., 2016 | Systematic review and meta-analysis of maternal, newborn, and child mHealth interventions in LMICs | Digital interventions have variable effects and cannot overcome absent transport or definitive emergency capacity. | Evaluate context-adapted mHealth for communication, reminders, consultation, and follow-up using service, equity, safety, and outcome measures. |

*Note: Older foundational sources were retained when they defined established frameworks or technical indicators. Reference 24 is a single illustrative case and is not used to infer prevalence. Abbreviations: ANC, antenatal care; BEmONC, basic emergency obstetric and newborn care; CEmONC, comprehensive emergency obstetric and newborn care; EmONC, emergency obstetric and newborn care; EPHS, Essential Package of Health Services; LMICs, low- and middle-income countries; mHealth, mobile health; MPDSR, maternal and perinatal death surveillance and response; WHO, World Health Organization.*
